# Studying immune to non-immune cell cross-talk using single-cell technologies

**DOI:** 10.1016/j.coisb.2019.10.005

**Published:** 2019-12

**Authors:** R. Elmentaite, S.A. Teichmann, E. Madissoon

**Affiliations:** 1 Wellcome Sanger Institute, Wellcome Genome Campus, Hinxton, CB10 1SA, United Kingdom; 2 Theory of Condensed Matter, Cavendish Laboratory, Department of Physics, University of Cambridge, Cambridge, CB3 0HE, United Kingdom; 3 European Molecular Biology Laboratory, European Bioinformatics Institute (EMBL-EBI), Wellcome Genome Campus, Hinxton, CB10 1SD, United Kingdom

**Keywords:** Single-cell technologies, Immunity, Cell-cell interactions, Cross-talk applications

## Abstract

Single-cell RNA-sequencing has uncovered immune heterogeneity, including novel cell types, states and lineages that have expanded our understanding of the immune system as a whole. More recently, studies involving both immune and non-immune cells have demonstrated the importance of immune microenvironment in development, homeostasis and disease. This review focuses on the single-cell studies mapping cell–cell interactions for variety of tissues in development, health and disease. In addition, we address the need to generate a comprehensive interaction map to answer fundamental questions in immunology as well as experimental and computational strategies required for this purpose.

## Highlights

• Systems view of immunology achieved via studying cell–cell interactions by single-cell methods.
• Importance of studying immune vs niche cell cross-talk in development, homeostasis and disease.
• Outlook of single-cell technologies in interrogating immune to niche cell cross-talk.

## Introduction

Understanding how a complex set of interacting components work together to produce an outcome requires a systems approach. For much of immunology's long history, most insights came from examination of the constituent parts and their individual characteristics and not so much on activities across the whole organism. This is understandable given the complexity of the immune system with hundreds of antigens, cytokines, chemokines and transcription factors that can be used to define a cell and its function. Single-cell technologies present an important strategy for understanding human immunity in a systematic way and have led to a major change in how research is performed in many laboratories. It is now feasible to use high-throughput methods for an unbiased exploration of a variety of circulating and tissue resident immune cells in health and disease, which has proven to generate new hypotheses. The ability to generate large single-cell atlases has evolved together with bioinformatic tools that enable the user to comprehend the vast amount of data produced [1]. To date, single-cell RNA-sequencing (sc-RNA-seq) has been successful at describing the heterogeneity of the immune system [2,3]. This has led to a discovery of novel circulating and tissue-resident immune cell types and states [4,5], reconstruction of cell lineages [6,7], and their activation status in disease [6,8]. Importantly, it has also revealed unexpected examples of cooperativity and partnerships between cell types [9].

Immune cells constantly survey tissues to monitor their integrity and respond to infections. Unsurprisingly, the greatest activity is found in border organs, including skin, lung and gut, that are all constantly exposed to physical and microbial stresses. In addition, there is a great functional and structural heterogeneity of resident immune cells across tissues. For example, liver macrophages called Kupffer cells specialise in the clearance of aged erythrocytes while gut macrophages regulate immune responses to commensal bacteria [10]. Even though the functional heterogeneity is likely dictated by tissue microenvironment, niche-specific factors involved are largely unknown [10]. That is because each organ is unique and tailored to suit the function of the tissue, containing a combination of specialised cell types from epithelial, endothelial, neural and mesenchymal lineages.

One way to interrogate immunology is by focusing not only on the immune cell types, but also on their non-immune interacting partners, molecules they use for communication and how such cross-talk changes during development, homeostasis and disease. The obvious advantage of single-cell technologies in addressing this task is the ability to sample various cell types in an unbiased way. Using the entire tissue allows not only the study of tissue-resident immune populations and their activation or differentiation stages but also their microenvironment. Here, we argue for the need of an integrative view of niche cells to achieve a systems view of immunology. We discuss increasing evidence that tissue cells communicate with resident and circulating immune cells to aid tissue development and regeneration, homeostasis and progression of disease. Finally, we end with a discussion of the existing experimental and computational strategies for dissecting this dialogue using single-cell technologies.

## Two sides of a coin: the roles of immune and their niche cells in immunity

**Figure 1:**
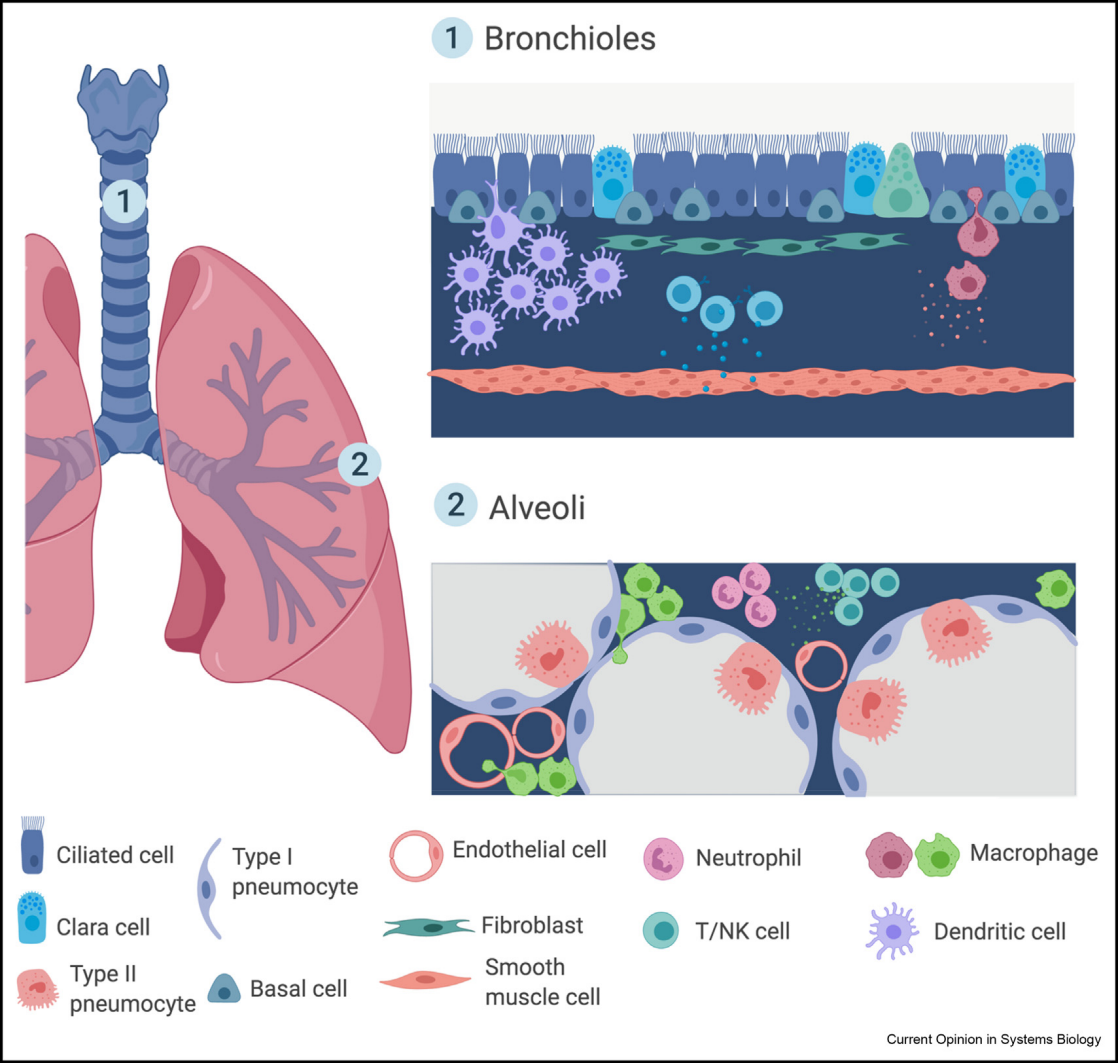
**Compartmentalisation of cell types and interactions within human lung.** Single-cell studies can outline the compartmentalisation, cellular content and cell–cell interactions within regions of a single organ. Cell–cell cross-talk will depend on the proportion of cells, proximity between different cell types and availability of interacting partners. Lung is an example of a highly structured organ with two main architectural locations: bronchioles and alveoli. Created with BioRender.com.

Cells can either interact physically via gap junctions and surface receptors or indirectly via secreted paracrine (local) and endocrine (distant) ligands. Predicting cell–cell communication from transcriptomics data is a challenging task. Nevertheless, multiple studies to date have attempted this task. The most common prediction approach is based on known ligand and receptor pairs and their expression on cells. The analysis requires a number of assumptions including that the receptor transcripts are translated and presented on the membrane, while ligands are successfully transported out of the cells, and are in proximity in tissue space with interacting partners. This is especially relevant to compartmentalised organs, such as lung, where immune and epithelial cell types and their proportions change drastically across multiple locations (Figure 1). Here, we highlight single-cell studies used to comprehensively delineate the functional cross-talk between immune and tissue niche cells and the relevance of these interactions in immunity and human disease.

### Development and tissue regeneration

Pregnancy presents an exceptional immunological problem, where the mother's immune system interacts with foetal cells in such a way that prevents rejection of ‘nonself’ and supports foetal growth and development. As shown by us and others, this subtle relationship is governed by the cross-talk between immune and non-immune cell types. An example of such interactions is where early embryonic epithelial trophoblasts express receptors that support maternal–foetal compartmentalisation and block the movement of inflammatory chemokines from the maternal decidua to embryonic circulation [11]. We [9] and others [12,13] have used single-cell technology to comprehensively infer complex interaction patterns during human first trimester and term pregnancies using receptor–ligand pairs. The studies inferred an interplay between maternal natural killer [9] and dendritic cells [13] with embryonic trophoblasts to achieve an anti-inflammatory environment.

The relationship seems to continue throughout adulthood and sustain tissue regeneration in multiple organs. Several studies report a tight cross-talk between tissue stem cells and immune cells as reviewed [14]. Single-cell studies offer unprecedented resolution in comprehensively identifying both the interacting partners and the molecules involved by using transcriptomics data. For example, a subset of T regulatory cells and macrophages was found to interact specifically with hair follicle stem cells [15,16]. Furthermore, both macrophages and ILC2 leukocytes expressing IL-13 were found to promote lung regeneration via modulation of alveolar stem cell self-renewal and differentiation [17]. Macrophages were found to contribute to gastric organoid regeneration *in vitro* [18] and in the murine intestine [19].

Single-cell approaches allow studying cell–cell interaction during mouse and human organogenesis. An example is a study in human foetal intestine, where CD4 Th1-like cells were shown to modulate intestinal growth via interaction with LGR5+ stem cells [20]. The potential of various immune cells including macrophages and basophils to interact with endothelial, fibroblast and epithelial cells was also shown in the developing murine lung [21].

### Homeostasis and infection

It is evident that tissue microenvironment changes drastically during infection, inflammation and mechanical injury. Single-cell studies have highlighted the structural and cellular compartmentalisation of various tissues relevant to responses in infections. In skin, fibroblast populations were compartmentalised into anti-inflammatory upper dermis and inflammatory lower dermis, which suggests that upper dermal fibroblasts are primed to respond to infection more readily [22]**.** Gene signatures in endothelial venule cells in peripheral lymph nodes [23] and skin fibroblasts [24] were described as consistent with recruitment of naive lymphocytes or retention of inflammatory cells, respectively. In addition, single-cell sequencing of murine lymph nodes has identified nine stromal cell populations that occupy multiple lymph node niches [25]. The study provides evidence that multiple stromal cell types contribute to the compartmentalised microenvironment, are in an activated state in a resting lymph node and guide immune cells during an immune response [25]. Furthermore, a subset of tuft cells from the gut epithelium was found to exhibit an inflammatory gene program with expression of Th2-promoting cytokine and immune cell marker Ptprc [26].

Moreover, the microenvironment can shape the immune cell differentiation potential. A single-cell study by Tikhonova et al. [27] described vascular, perivascular and osteoblast cells in the adult bone marrow. They found that Notch ligand DLL4 expressed by endothelial cells skews differentiation of hematopoietic progenitors to the myeloid lineage. Conversely, a single-cell study of mouse skin during wound healing has identified a subset of myofibroblasts and rare regenerated adipocytes that have originated from myeloid cells [28]. Pseudotime and RNA velocity analyses revealed a subset of contractile fibroblasts that expressed hematopoietic markers and validated that cells originating from the bone marrow give rise to a subset of myofibroblasts and rare regenerated adipocytes during wound healing. Similarly, immune cells can shape epithelial cell differentiation in inflammation. In mice, that has been observed upon helminth and bacterial infection, which results in specialisation of intestinal epithelial cells to different secretory lineages [26,30].

### Disease and aging

The immune microenvironment has received a lot of attention in cancer and has been the subject of multiple reviews [31,32]. Single-cell studies have contributed by identifying specific T-cell [33,34] and macrophage [35,36] populations that are predictive of the clinical outcome in lung cancer and melanoma. Furthermore, the spatial distribution of a T-cell subset around the malignant cells was important for the outcome in B-cell lymphoma [37]. These studies outline potential of single-cell profiling tools in both the diagnostics in cancer as well as for development of therapeutics.

Niche cell populations can also shape immune cell function in other human diseases and aging. For example, inflammatory diseases can manifest as a result of imbalanced immune cell recruitment or retention modulated by niche cell signalling. Inflammation-related keratinocyte signatures were enriched in psoriatic skin, alongside increased numbers of a specific *CD1C*+*CD301A*+myeloid dendritic cell population [38], pointing to a potential recruitment function of niche cells. Also, fibroblasts were associated with recruitment of macrophages [39] and T-cells [40] in melanoma and with retention of T cells in inflamed human skin [41]. Moreover, there has been increased attention on fibroblast populations in inflammatory bowel disease (IBD), rheumatoid arthritis (RA) and cancer. Single-cell study in healthy and IBD patients identified four populations of fibroblast-like cells, one of which was enriched for pro-inflammatory factors and expanded in IBD [42]. Similarly, a population of fibroblasts in the joint sublining layer were associated with severity of inflammation in RA patients [43,44]. Fibroblast invasiveness was further found to be induced via a newly identified subset of macrophages in RA [45].

The relationship between chronic inflammation and aging has been reported and reviewed elsewhere [46,47]; however, the mechanisms are currently unknown. Single-cell studies show higher mutational burden and increasing heterogeneity with age for multiple cell types as well as upregulation of inflammatory signatures both in immune and non-immune cells [48,49]. The combination of having more cytokine-secreting fibroblasts [50] and heterogeneous activation of lymphocytes [50,51] in old mice versus young suggest an increasingly versatile cross-talk between cells in an aging organism. This emphasises the need to study age-related conditions and cell–cell interactions via single-cell methods.

## Single-cell strategies for profiling cell–cell communication

To use sc-RNA-seq data to comprehensively map all immune to non-immune cell interactions, there is a need to optimise the unbiased capture of all cell types in single-cell experiments. Indeed, the unbiased sampling of organs to date has highlighted previously unappreciated diversity in immune and non-immune cell types in healthy human lung [8,52], liver [53], colon [42,54], skin [38,55] and pancreas [56,57]. However, the unbiased sampling requires sequencing of large numbers of cells. This increases the cost of a study, especially if one major cell type dominates over others. Also, not all cell types respond equally to the same sample processing protocol, with some cell types requiring harsh dissociation methods that are likely to kill other cell types. For example, structural parts of a tissue, like smooth muscle cells or enteric neuron networks in the gut, will require longer and harsher dissociation than epithelial cells of the lamina propria. Therefore, the same tissue might have to be sampled using different dissociation protocols to cover the diversity of cell types present.

To down-sample abundant cell types, enrichment of cell lineages can be done to ensure balanced cell capture. For example, less abundant populations can be sorted using a marker gene, such as immune (CD45RA), epithelial (EPCAM) or fibroblast (COL1A1) cells, which can be combined with the remaining cells in desired proportions [9,40,58]. This approach leads to datasets with better balance of different cell types and reduction in sequencing costs. However, the downsides include a longer manipulation time for cell enrichment that might affect cell viability or induce stress response gene expression [59]. Finally, it may also be of interest to interrogate the signalling of two known, but rare interacting partners. In this case, classical isolation and in-depth sequencing of underrepresented cell population could be used combined with unbiased sampling. To date, the combination of single-cell methods with other approaches have predicted cell–cell proximity such as using partial dissociation and downstream de-convolution of the signal [60] or cell lineage labelling methods such as NICHE-seq [61].

## Outlook and summary

In single-cell experimental design, there is a trade-off between in-depth profiling of specific cell types and unbiased mapping of all cell types present in the tissue. Apart from describing immune heterogeneity, it has provided a systems insight into immunology during development, regeneration, infection and disease.

**Figure 2:**
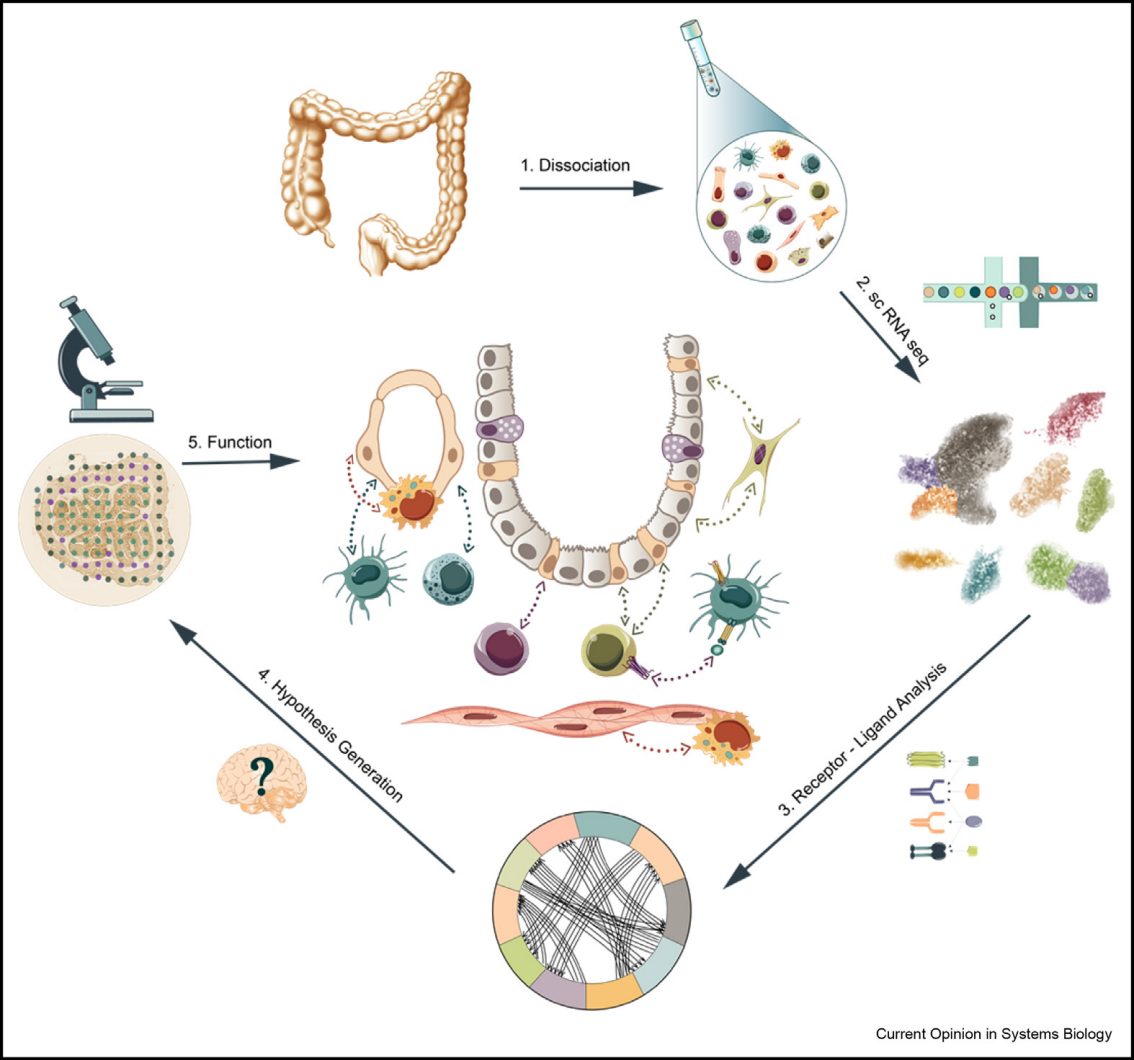
**Schematic workflow for analysing cell–cell interactions.** Tissue sample is dissociated and analysed by sc-RNA-seq followed by cell-type identification. Previous knowledge of receptor–ligand pairs is used for inferring cell–cell communication and formulating new hypotheses, which are validated by molecular biology methods, thus providing a model for the interaction network within a sampling location.

As evident from the studies discussed above, single-cell methods alone can be extremely informative in providing cell–cell interaction snapshots in human tissues (Figure 2). The publicly available receptor–ligand inference tools such as CellPhoneDB [9] and ProximID [60] are user friendly and easily applicable. However, major disadvantages of sc-RNA-seq include loss of spatial information including cell–cell proximity as well as the distribution of signal within a cell, especially in highly structured cell types such as neurons and dendritic cells. Therefore, there is a need for high-resolution spatial methods that can capture the full transcriptome *in situ*. Although traditional microscopy, with either use of antibodies [37] or RNA probes [62], provides subcellular resolution, a very limited set of features per cell can be studied. High-throughput spatial genomics methods aim to characterise gene expression from single cells while retaining information on tissue context. The methods of hybridisation by MERFISH [63] or sequencing by FISSEQ [64] on slides captures expression of up to thousands of genes and their subcellular locations. Although Spatial Transcriptomics methods [65] allow for the complete transcriptomic profile of a tissue region containing a dozen to hundreds of cells, a higher density array such as Slide-seq [63] allows for subcellular localisation with up to multiple cellular localisations captured. Currently, none of these methods match sc-RNA-seq in price, accuracy, depth, robustness and number of cells gained. However, we anticipate that if the methods combining spatial subcellular resolution and transcriptomics improve, they will be very informative and widely used in the future.

As the Human Cell Atlas (www.humancellatlas.org) initiative is working on describing all cell types in the human body, the meta-analysis of cell–cell interaction networks in multiple tissues will provide another level of insight, including the inference of paracrine signalling. In the future, deconvolution of interactions using multi-omics including genomics, proteomics, metabolomics and gene sequencing data will be the key to better understand the downstream signalling and correlations among genes, proteins and lipids at the single-cell level. As the repository of all possible interactions and downstream signalling increases, so will the ability to interpret the interactions between single cells.

**Figure 3:**
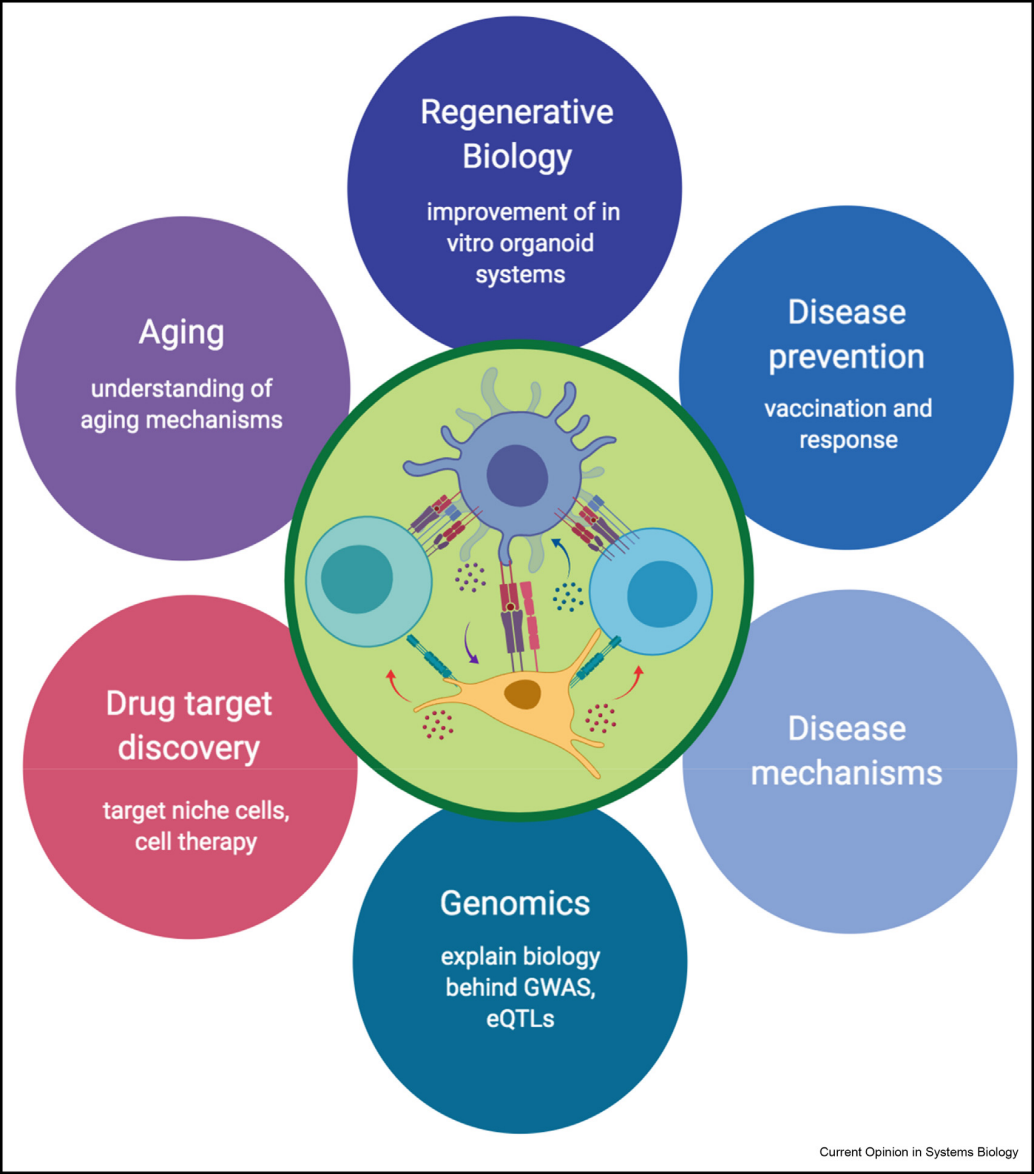
**Applications of cell–cell interaction networks in science and medicine.** Studies of interaction networks in the single-cell resolution will advance both basic and applied research. Better understanding of regenerative biology, aging and genetic vs environment relationship will lead to better cell therapies and discovery of new drug targets. Created with BioRender.com.

Better understanding of cell–cell interactions will inevitably lead to multiple applications (Figure 3) [66]. For example, culture conditions can be inferred from newly defined signalling pathways. This will have applications in improving *in vitro* differentiation processes and regenerative biology [67]. Cellular identity of the interacting partners in disease will provide new candidates for cell therapies and enhance the effectiveness of existing ones [8,68]. Furthermore, interactions may aid in understanding and predicting tissue and cell type specific efficacy of drugs and vaccinations as well as their side-effects. With more data becoming available, the ability to explain environmental and genetic effects on cancer, drug response, chronic inflammation and others will become a reality.

## Conflict of interest statement

Nothing declared.
